# Gender Differences in Service Utilization and Pattern of Free Presbyopia Glasses Distribution among Patients at Eye Camps in Nepal: A Retrospective Study

**DOI:** 10.31729/jnma.9109

**Published:** 2025-06-30

**Authors:** Ranjan Shah, Sailesh Kumar Mishra, Pathiyil Ravi Shankar, Anup Subedi, Prakriti Sharma, Anil Paudel, Pankaj Ray Adhikari, Amit Kumar Singh

**Affiliations:** 1 Nepal Netra Jyoti Sangh, Central Office, Tripureshwor, Kathmandu, Nepal; 2 IMU Centre for Education, IMU University, Kuala Lumpur, Malaysia; 3 Himalaya Eye Hospital, Nepal Netra Jyoti Sangh, Gharipatan, Pokhara, Nepal; 4 Nepal Health Research Council, Ram Shah Path, Kathmandu, Nepal; 5 Department of Public Health, CiST College, Kathmandu, Nepal; 6 Biratnagar Eye Hospital, Biratnagar, Nepal; 7 Mechi Eye Hospital, Birtamod, Jhapa, Nepal.

**Keywords:** *eye care*, *gender-difference*, *Nepal*, *presbyopia*, *service-utilization*

## Abstract

**Introduction:**

Presbyopia is an age-related refractive condition that causes difficulty in seeing nearby objects clearly. It affects a significant portion of the world's population, especially individuals over 40 years old. Many studies show variation in the utilization of eye healthcare services according to gender in resource-limited settings like Nepal. This influence access to eye care service. This study aims to investigate gender-based differences in service utilization and prescription patterns at free presbyopia camps in Nepal.

**Methods:**

A retrospective observational study analyzed anonymized socio-demographic and clinical data of 29,943 presbyopic patients attending free eye camps organized by twenty-six NNJS eye hospitals/centers across Nepal from 2020 to 2024. Ethical approval was obtained from the Institutional Review Committee of Nepal Netra Jyoti Sangh. Data on age, gender, and spectacle prescriptions were collected to assess service utilization and gender disparities.

**Results:**

Among the total presbyopic patients 15,356 (51.3%) had high presbyopia, with a slightly higher prevalence among males 8,025 (52.3%) compared to females 7,331 (47.7%). Presbyopia prevalence increased with age, peaking at 12,522 (41.8%) in those >56 years. Females had higher prevalence in younger age groups, while males predominated in older groups.

**Conclusions:**

In this study, presbyopia was common in age group >56 years with service utilisation more by male.

## INTRODUCTION

Presbyopia is an age-related refractive condition affecting the ability to see nearer objects clearly. Globally it affects 1.8 billion people, while at least 2.2 billion people have near or distance vision impairment.^1^ It is prevalent among middle-aged adults.^2^ Gender inequality can restrict healthcare access, with women in South Asia and Nepal frequently encountering more obstacles due to cultural norms, economic reliance, and limited mobility which affect their use of services like presbyopia treatment.^3,4^

More than 80% of individuals with presbyopia experienced near vision problems with women reporting similar dissatisfaction as men with their ability to perform near tasks.^5,6^ In Nepal, a study found overall prevalence of 59.7%, in individuals aged 35 years and above and with the highest rate of 86.8% observed in those aged 50-59 years.^7^

Existing literature suggests that there are disparities in seeking health services between males and females. Therefore, this study examines the gender differences in service utilization and prescription patterns among patients at a free presbyopia glass distribution camp.

## METHODS

### Study design and setting

This retrospective observational study utilized data from free eye camps organized by twenty-six eye hospitals and eye care centers under Nepal Netra Jyoti Sangh (NNJS) across seven provinces of Nepal between 2020 and 2024. These camps provided free surgical services, medicines, and presbyopic glasses to patients. Socio-demographic and clinical data of presbyopic patients attending the camps were collected anonymously using a standard proforma and analyzed retrospectively.

Approximately 42,500 patients benefited from these camps, among whom 29,943 (70.1%) were diagnosed with presbyopia. Ethical approval was obtained from the Institutional Review Committee of NNJS (Reference Number: 092/081/82), adhering to the Declaration of Helsinki. A letter of undertaking was also secured from NNJS for the use of camp data. The study included secondary data of individuals with near-vision problems who sought presbyopia glasses during the eye camps. The primary objective was to characterize the clinical features and demographics of individuals diagnosed with presbyopia in these free eye camps.

### Examination protocol

With prior approval from the respective local municipality and in coordination with local organizers, surgical eye camps or diagnostic, screening and treatment (DST) eye camps were conducted by a hospital team led by an ophthalmologist. Distant visual acuity (VA) was measured using a Snellen chart from 6-meter distance. Participants with age 35 years and above having 6/12 or better distance visual acuity underwent refraction for near vision, while individuals having visual acuity less than 6/12 were referred for refraction for distance vision. Near visual acuity was assessed with and without presenting near correction at a distance of 40 cm using a reduced Snellen near vision tumbling E chart under standard indoor lighting, with a cut-off of reduced Snellen 6/9 or better. Those participants who had uncorrected binocular distance vision of ≤ 6/12 underwent refraction for distance, and participants with near vision of ≤ 6/9 were tested with plus spheres to establish the best-corrected binocular near vision acuity. Participants who were presented with due binocular near vision of ≤ 6/9 but improved with plus lenses were provided near vision glasses at no cost. Glasses were dispensed according to the accommodative demand and need of the participants.

### Data management and statistical analysis

Data captured initially in Microsoft Excel were processed and exported to Statistical Package for the Social Sciences version 25 for analysis. Descriptive analysis such as frequency distributions and percentages were used to summarize demographic and clinical characteristics. The primary outcomes were calculated using 95% confidence intervals (CIs) in order to ensure reliability of the results. The analysis was aimed at comparing service use and prescription patterns for men and women and identifying possible associations with age.

## RESULTS

A total of 29,943 participants were enrolled in the study, of which 15,357 (51.29%) were females.

The mean age of the participant was 54.87±10.71 years. There were 8,384 (28%) particpants from Lumbini province, 7,249 (24.21%) from Bagmati 4,910 (16.40%) from Gandaki. A total of 14,717 (49.15%) patients were examined by NNJS (Table 1).

**Table 1:** Demographic characteristics of the participants

Demographic characteristics		Frequency (%)
Province	Bagmati	7249 (24.21)
	Far Western	664 (2.22)
	Gandaki	4910 (16.40)
	Karnali	2919 (9.75)
	Koshi	3751 (12.53)
	Lumbini	8384 (28.00)
	Madhesh	2066 (6.90)
Eye camp Organised by	Eye care center	4551 (15.20)
	Vertical project	2910 (9.72)
	Secondary eye hospital	7765 (25.93)
	Tertiary eye hospital	14717 (49.15)
Age category	35-39	689 (2.30)
	40-45	6138 (20.50)
	46-50	5351 (17.87)
	51-55	5279 (17.63)
	>56	12486 (41.70)

There were 5,837 (38.01%) female and 6,709 (45.99%) male with presbiopia in age group ≥56 years. (Table 2).

**Table 2:** Distribution of presbyopic patients by age and gender

Age/Gender	Female N (%)	Male N (%)
35-39	384 (2.50)	322 (2.21)
40-45	3626 (23.61)	2523 (17.30)
46-50	2864 (18.65)	2421 (16.60)
51-55	2646 (17.23)	2611 (17.90)
≥56	5837 (38.01)	6709 (45.99)

**Table 3:** Distribution of presbyopia by severity

Amount of presbyopia	Female N(%)	Male N(%)	Total N(%)
Low (+0.25 to +0.75 DS)	16 (59.3)	11 (40.7)	27 (0.1)
Moderate (+1.00 to +2.00 DS)	8010 (55.0)	6550 (45.0)	14560 (48.6)
High (+2.25 to +3.00 DS)	7331 (47.7)	8025 (52.3)	15356 (51.3)
Total	15357 (51.3)	14586 (48.7)	29943 (100.0)

**Table 4:** Relation between age and power

Age category (yrs)	Power (mean ±SD)
35-39	1.20±0.528
40-45	1.35±0.387
46-50	1.85±0.373
51-55	2.26±0.344
≥56	2.74±0.414

**Distribution of presbyopia by severity**

Among individuals with low presbyopia, female accounted for 16 (59.25%), while male comprised 11 (40.75%) (Table 3).

The participants aged 35-39 years required lower power lenses (mean = +1.20 DS), while older participants (>56 years) required higher power (mean = +2.74 DS) (Table 4).

## DISCUSSION

The study analyzed data from the participants attending presbyopia screening camps in Nepal, examining demographic distribution, gender differences and variation in spectacles power. The findings of the study showed predominance of females 15,357 (51.29%), significant gender disparities in age distribution and higher spectacles power requirement in older males. The gender disparity may be attributed to factors such as women's limited access to fixed facilities, financial constraints, and their responsibilities in household tasks. These barriers are mitigated by the convenience of eye camps conducted at their doorstep. The findings of this study support previous research showing gender-based access gaps in eye care, particularly in low-resource settings, and emphasize the impact of age and gender on healthcare consumption.^8,9^ Similarly, from the study in Tanzania states, high percentage of female are more likely to suffer from presbyopia from age 40 and above then men.^6^

In the present study, there were more male in the older age groups. According to earlier research, men in older age groups tend to use healthcare services more frequently because presbyopia and other age-related illnesses are more common in them.^10^ However, the overall percentage was somewhat higher for women, which is consistent with broader public health research showing that women are more likely than men to seek medical assistance at a younger age.^11^ These results emphasize how crucial gender-sensitive planning and execution strategies are for community eye health initiatives.

Furthermore, gender disparities were observed in the distribution of corrective lens powers, with males more often prescribed higher powers, especially +2.50 D and above (52.29% in males and 47.71% in females) which indicates greater attendance of older males in health camps in comparison to females. Likewise, in earlier research observed men frequently need heavier prescriptions because they are less likely to seek early vision treatment.^10,12^ The findings highlight the importance of incorporating gender-based variations into eye care programs to enhance the effectiveness of these health camps and optimize service delivery.

The findings of the study highlight the importance of incorporating age and gender sensitivity into outreach initiatives. It provides foundation for designing vision care camps that address the specific needs of diverse populations, thereby enhancing access and potentially improving long-term vision outcomes for at-risk groups. Future research could build on these findings to develop comprehensive strategies for delivering equitable eye care services to similar populations.^13,14^ slit lamp biomicroscopy of the anterior segment, and dilated posterior segment examinations were performed using a standardized protocol for subjects identified through a random cluster-sampling strategy in Andhra Pradesh. Information of difficulty in performing near tasks was collected as part of a visual function questionnaire administered to all subjects. A person was defined as having presbyopia if the person required an addition of at least 1.0 D in either eye for near vision in addition to their best corrected distance correction to improve near vision to at least N8 and if they had graded lens opacities (Lens Opacities Classification System [LOCS III] system.

In the current study, retrospective design limits the ability to establish causation, and the data might contain selection biases. Additionally, the lack of socioeconomic and geographic data prevents more comprehensive analysis of external variable that could affect prescription patterns and service consumption.^8,15,16^ More thorough strategy that takes these factors into account could help future studies to gain a better understanding of how presbyopia services are delivered in environments with limited resources.

This study has implications for clinical practice as well as future research. The result of this study suggests that health camps can be more effective if strategies are tailored to meet age and gender specific needs. Additionally, demographic projections indicate the need for camps to adjust the variety and quantity of lenses available to accommodate the differing power requirements across genders^17-20^ It is advised that future longitudinal research investigate the underlying reasons of these trends and how they evolve over time in order to improve intervention techniques and optimize the effectiveness of eye care camps.

## CONCLUSIONS

In this study, presbyopia was common in age group >56 years with increase in power after 50 years. The study showed male predominance in service utilisation along and severity was more in male, which was consistent with other studies.
